# Genetic Architecture of Synaptic Failure in Dementia with Lewy Bodies: From α-Synuclein Proteoforms to GBA1-Mediated Plasticity Deficits

**DOI:** 10.3390/genes17080965

**Published:** 2026-08-18

**Authors:** Anastasia Bougea

**Affiliations:** 1st Department of Neurology, Eginition Hospital, National and Kapodistrian University of Athens, 11528 Athens, Greece; abougea@med.uoa.gr

**Keywords:** dementia with Lewy bodies, α-synuclein, *GBA1*, glucocerebrosidase, synaptic plasticity, long-term potentiation, proteoforms, synaptopathy, neuropsychiatric symptoms, gene therapy, disease modifiers

## Abstract

Dementia with Lewy bodies (DLB) is increasingly conceptualised not merely as a disorder of neuronal death but as a primary synaptopathy in which the functional collapse of synaptic transmission and plasticity precedes, and predicts, neurodegeneration and clinical decline. Two genetic determinants dominate the heritable risk architecture of DLB: the α-synuclein gene *SNCA*, in which both copy-number variation and missense mutations exert dose- and conformation-dependent effects, and GBA1, encoding the lysosomal hydrolase glucocerebrosidase (GCase), the single most influential genetic risk factor for the disease. Here we synthesise evidence that these loci converge on a shared pathogenic endpoint—the impairment of activity-dependent synaptic plasticity. We argue that *GBA1* loss-of-function and the resulting accumulation of glucosylceramide stabilise specific neurotoxic α-synuclein proteoforms, including soluble oligomers and self-templating conformational strains bearing defined post-translational modifications. These proteoforms are trafficked to, and enriched within, presynaptic terminals, where they disrupt SNARE-complex assembly and synaptic-vesicle dynamics, while postsynaptically they perturb NMDA and AMPA receptor trafficking, dysregulate dendritic calcium, and compromise synaptic mitochondrial bioenergetics. The net consequence is a metaplastic shift away from long-term potentiation (LTP) and toward aberrant long-term depression (LTD), a signature of synaptic failure detectable before frank pathology. We map these molecular events onto disease-relevant circuits—particularly the cholinergic basal forebrain and hippocampal–cortical and thalamocortical networks—and relate them to the defining neuropsychiatric features of DLB, including cognitive fluctuations and recurrent visual hallucinations. Finally, we evaluate emerging therapeutic strategies that target the GBA1–α-synuclein axis and that aim to restore synaptic plasticity directly. Positioning DLB within the framework of genetically determined plasticity deficits clarifies its kinship with other neuropsychiatric disorders and identifies the synapse as the most tractable node for early, disease-modifying intervention. We further examine how GBA1 allele severity and zygosity grade the phenotype, which genetic and environmental factors modify penetrance in carriers, and what distinguishes this synaptopathy from those driven by PSEN1/PSEN2, MAPT, or HTT, and we summarise the therapeutic pipeline—including enzyme augmentation and adeno-associated viral GBA1 gene therapy—that targets it.

## 1. Introduction

Dementia with Lewy bodies sits at the intersection of neurodegenerative and neuropsychiatric disease. Clinically, it is defined by the fourth consensus criteria of the DLB Consortium around a tetrad of core features—fluctuating cognition, recurrent well-formed visual hallucinations, rapid eye movement sleep behaviour disorder, and spontaneous parkinsonism—superimposed on a progressive dementia [1]. Neuropathologically, it is characterised by intraneuronal accumulation of misfolded α-synuclein into Lewy bodies and Lewy neurites, frequently co-occurring with Alzheimer-type co-pathology [2,3]. Yet the conventional emphasis on inclusion bodies and cell loss has obscured a more proximal and arguably more therapeutically relevant lesion: the early and selective failure of the synapse.

The genetic landscape of DLB, though more sparsely characterised than that of Parkinson’s disease (PD), points repeatedly toward synaptic biology. Genome-wide association and sequencing studies have established SNCA and GBA1 as the principal risk loci, with the APOE ε4 allele contributing additional, partly amyloid-dependent risk [4,5,6]. Strikingly, the two leading genetic drivers encode proteins whose physiological functions and pathological dysfunctions are anchored at the presynaptic terminal and the lysosome–autophagy system that maintains it. This convergence motivates a reframing of DLB as a genetically determined synaptopathy [7].

The synaptopathy hypothesis holds that the earliest measurable deficit in DLB is not neuronal death but a loss of synaptic integrity and function—reductions in synaptic density, impaired neurotransmitter release, and a breakdown of the activity-dependent plasticity that underlies learning, memory, and the dynamic regulation of cortical networks [7,8]. Quantitative neuropathology has shown that aggregated α-synuclein accumulates predominantly at presynaptic terminals rather than within the somatic Lewy body and that this presynaptic burden correlates with dendritic spine loss and cognitive impairment more closely than does inclusion-body count [8,9]. Convergent work positions synaptic dysfunction, rather than overt degeneration, as the initiating event in synucleinopathy [10].

**Figure 1:**
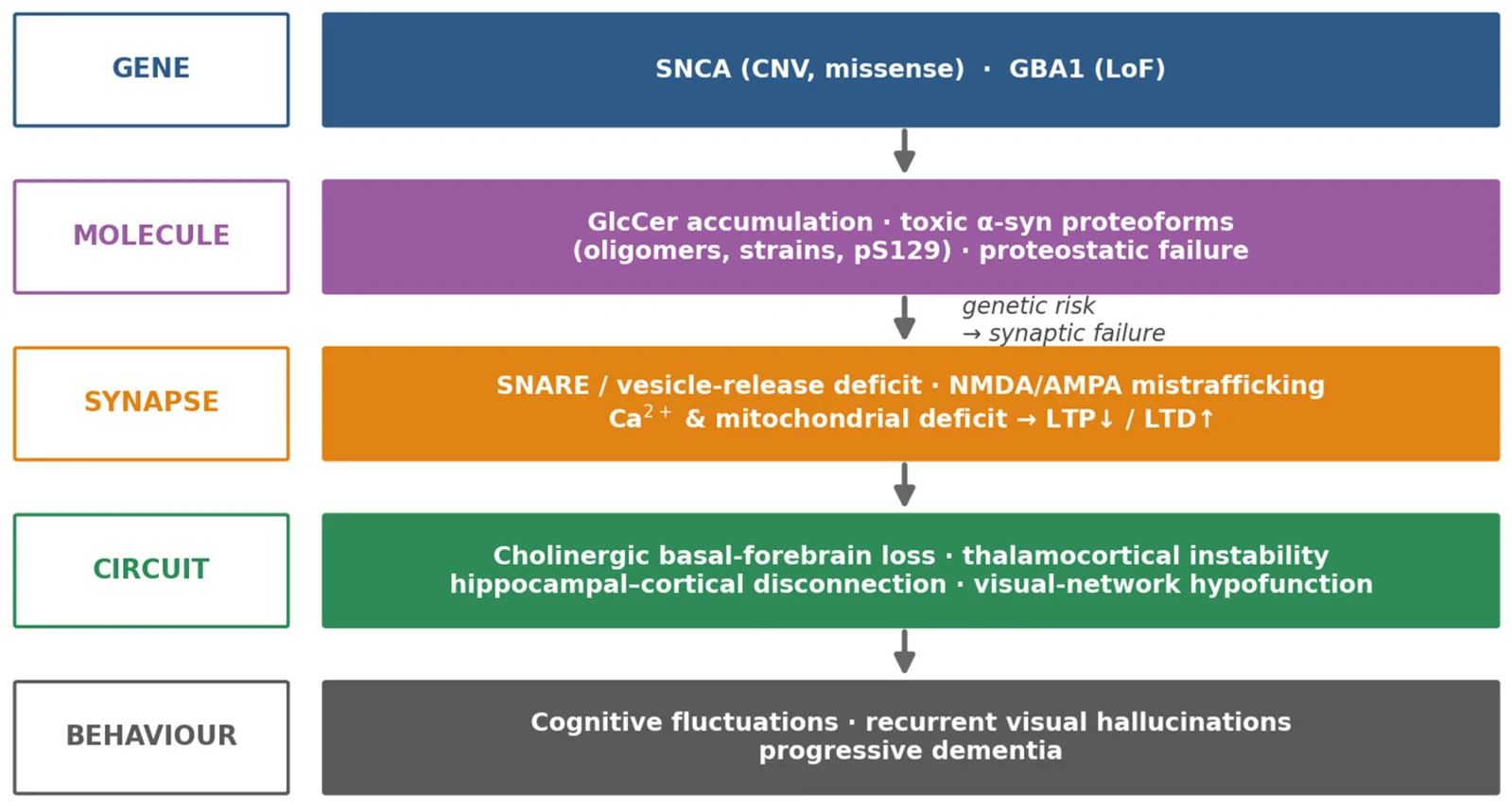
Multi-level integration from genotype to neuropsychiatric phenotype. Genetic risk (SNCA dosage and missense variants; GBA1 loss-of-function) determines the molecular load and identity of α-synuclein proteoforms and the lysosomal lipid environment. These act at the synapse to disrupt release machinery and glutamate receptor trafficking, producing an LTP/LTD imbalance that propagates to cholinergic, thalamocortical, and hippocampal–cortical circuits, generating the cognitive fluctuations, recurrent visual hallucinations, and progressive dementia that define the clinical syndrome.

This review aligns DLB with the central theme of this Special Issue—the genetic mechanisms of synaptic plasticity in neuropsychiatric disorders—by tracing an explicit causal arc from gene to circuit to behaviour. We move from the discovery of risk loci, through the molecular machinery by which GBA1 deficiency generates pathogenic α-synuclein proteoforms, to the biophysics of impaired LTP and aberrant LTD, and finally to the circuit-level disturbances that manifest as the neuropsychiatric phenotype of DLB (Figure 1).

## 2. The Genetic Landscape of Synaptic Vulnerability in DLB

### 2.1. SNCA: Dosage, Conformation, and the Primacy of the Presynaptic Protein

α-Synuclein, encoded by SNCA on chromosome 4q22, is a small, natively disordered, presynaptically enriched protein whose physiological roles are intimately tied to synaptic-vesicle biology; it chaperones SNARE-complex assembly through direct interaction with VAMP2/synaptobrevin-2 [11]. The genetic evidence implicating SNCA in Lewy body disease operates through two distinct mechanisms. Copy-number variants cause disease in a strict gene-dosage-dependent manner: duplications typically produce later-onset parkinsonism with variable cognitive involvement, whereas triplications, doubling protein expression, yield earlier onset, prominent autonomic failure, and a markedly higher burden of dementia and psychosis resembling the DLB end of the spectrum [12,13]. Comparative analysis of multiplication kindreds confirmed a direct relationship between SNCA dosage and the severity of cognitive and neuropsychiatric decline [14]. This dose–phenotype relationship establishes a fundamental principle: the quantity of α-synuclein available at the synapse is itself a determinant of neuropsychiatric severity.

Missense mutations act through a complementary, conformation-based mechanism, altering aggregation kinetics, membrane affinity, and fibril identity. The first to be identified, *A53T*, was followed by *A30P*, *E46K*, *H50Q*, *G51D*, and *A53E*; several of these accelerate the formation of the soluble oligomeric intermediates most closely associated with synaptic toxicity, and *E46K* in particular was described in a kindred with Lewy body dementia [15,16,17]. Common non-coding SNCA polymorphisms identified by association studies, which modestly elevate expression, confer the bulk of population-level risk and reinforce the dosage logic derived from the rare multiplication families [18].

### 2.2. GBA1: The Dominant Modifier of Synaptic and Cognitive Trajectory

Heterozygous variants in *GBA1* (chromosome 1q21), encoding glucocerebrosidase, constitute the most common and most consequential genetic risk factor for DLB, with odds ratios exceeding those of any other locus [19]. Biallelic GBA1 mutations cause Gaucher disease, and the recognition that carriers show elevated rates of parkinsonism and dementia first linked sphingolipid metabolism to synucleinopathy [19]. A dedicated multicentre analysis confirmed enrichment of GBA1 variants in DLB specifically, and this enrichment was associated with earlier onset and a more florid neuropsychiatric presentation [20]. GBA1 variants stratify by severity in a manner that maps onto outcome: severe alleles (e.g., p.L483P) confer greater risk and faster cognitive decline than mild alleles (e.g., p.N409S) [21,22].

**Figure 2:**
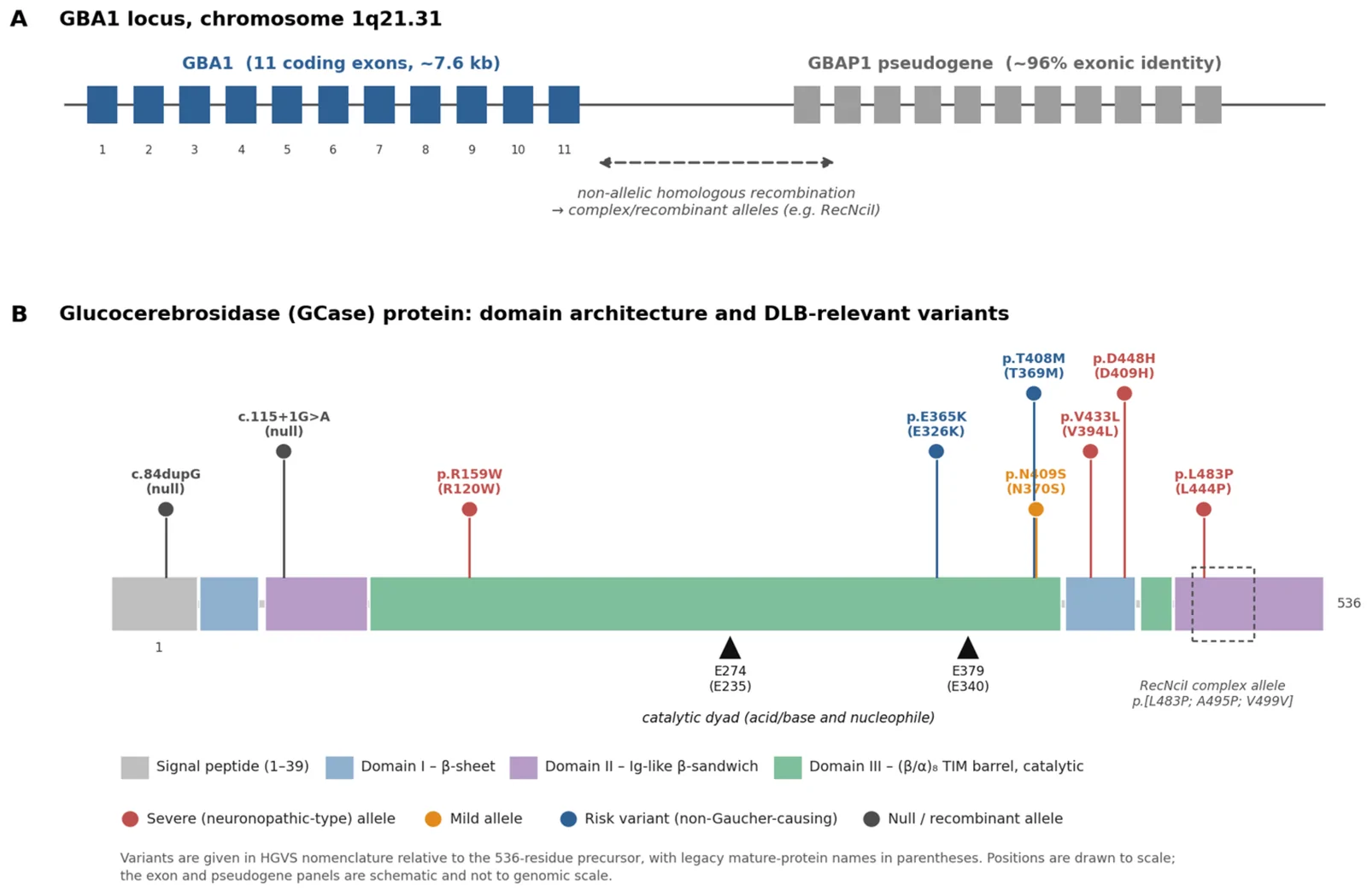
Architecture of the *GBA1* locus and of glucocerebrosidase, with the position and severity class of the variants most relevant to dementia with Lewy bodies. (**A**) *GBA1* comprises eleven coding exons at chromosome 1q21.31 and lies immediately telomeric to the highly homologous GBAP1 pseudogene; non-allelic homologous recombination between the two generates complex or recombinant alleles such as RecNciI. (**B**) The 536-residue precursor contains a 39-residue signal peptide followed by three domains: domain I (β-sheet), domain II (immunoglobulin-like β-sandwich), and domain III, the (β/α)8 TIM barrel that harbours the catalytic dyad (Glu274 and Glu379; legacy Glu235 and Glu340). Lollipops mark representative variants coloured by severity class: severe (neuronopathic-type) alleles, the mild p.N409S allele, non-Gaucher-causing risk variants, and null or recombinant alleles. Variants are given in HGVS nomenclature relative to the precursor protein, with legacy mature-protein names in parentheses. Residue positions are drawn to scale; the exon and pseudogene panels are schematic and not to genomic scale.

The structural basis of this allelic hierarchy is informative, and it is summarised in Figure 2. *GBA1* comprises eleven coding exons at 1q21.31 and lies approximately 16 kb telomeric to a highly homologous pseudogene, GBAP1, whose exonic sequence is about 96% identical. Non-allelic homologous recombination between the two generates the complex and recombinant alleles, such as RecNciI, that behave as severe low-activity variants and that are systematically missed by short-read sequencing pipelines unless *GBA1* is interrogated specifically [19,21]. The 536-residue precursor is processed to a 497-residue mature enzyme organised into three domains: an amino-terminal β-sheet domain (domain I), an immunoglobulin-like β-sandwich (domain II), and a (β/α)8 TIM barrel (domain III) that carries the catalytic dyad Glu274 and Glu379 (legacy Glu235 and Glu340) [23,24]. Severe alleles such as p.L483P (legacy L444P) map to the hydrophobic core of domain II and destabilise the folded protein, whereas the mild p.N409S (N370S) allele lies close to the catalytic barrel and impairs catalysis and lysosomal stability rather than global folding. The non-Gaucher-causing risk variants p.E365K (*E326K*) and p.T408M (*T369M*) reduce activity only modestly, yet they carry disproportionate cognitive risk, which indicates that the cognitive phenotype is not a simple linear function of residual catalytic activity [21,22,25,26].

### 2.3. Allele Dosage and Zygosity: How GBA1 Genotype Shapes the DLB Phenotype

*GBA1* behaves neither as a classical recessive nor as a classical dominant locus, and the phenotypic consequences of zygosity are asymmetric. In the heterozygous state, which accounts for the overwhelming majority of GBA1-related Lewy body disease, residual GCase activity is approximately half of wild-type in leucocytes and in brain, and this partial deficiency is sufficient to shift the α-synuclein proteoform equilibrium without producing systemic Gaucher disease [23,27]. Heterozygous carriers show a several-fold increase in the odds of DLB, and in the multicentre series that examined DLB directly, the effect size exceeded that reported for Parkinson’s disease [19,20]. Mediation analysis in post-mortem cohorts indicates that this effect is transmitted principally through an increase in the burden of mutant α-synuclein species rather than through an independent lysosomal pathway [28].

Biallelic genotypes, which cause Gaucher disease, magnify this risk rather than change it qualitatively. In the International Collaborative Gaucher Group registry, patients with type 1 Gaucher disease developed parkinsonism far more often than population expectation and one to two decades earlier than in sporadic disease [29]. Longitudinal deep phenotyping of *GBA1* pedigrees showed that homozygotes and compound heterozygotes accumulated prodromal markers—hyposmia, rapid eye movement sleep behaviour disorder, and subtle cognitive slowing—earlier and more frequently than their heterozygous relatives, who in turn exceeded non-carrier controls [30]. The gradient is therefore quantitative: two mutant alleles confer earlier and more penetrant disease than one, and one confers substantially more risk than none.

Allele severity, however, dominates zygosity as a predictor of the cognitive phenotype. Heterozygosity for a severe allele (p.L483P, p.D448H, or a recombinant allele) is associated with faster cognitive decline, earlier dementia, and more florid psychosis than heterozygosity for the mild p.N409S allele [21,22]. The risk variants illustrate the same principle from the opposite direction: p.E365K does not cause Gaucher disease even in the homozygous state, yet carriers show accelerated cognitive decline and a distinctive amnestic-plus-visuospatial profile [25,26,31]. The most parsimonious interpretation is that the phenotype tracks the integrated lifetime deficit in lysosomal GCase activity and the resulting glucosylceramide burden—an effectively continuous variable set jointly by the number of mutant alleles, the residual activity that each allele encodes, and the modifiers of the remaining wild-type allele—rather than by zygosity as such.

Two caveats temper this synthesis for DLB specifically. First, almost all dose–phenotype data derive from Parkinson’s disease and Gaucher disease cohorts; the DLB series are small, and biallelic DLB is rare enough that no adequately powered comparison of homozygous with heterozygous DLB has been published. Second, pseudogene-derived complex alleles are systematically under-ascertained by standard genotyping, so the nominally heterozygous category almost certainly contains cryptic severe genotypes [19]. Prospective DLB cohorts genotyped with long-read or GBA1-specific assays, and stratified by predicted residual enzyme activity rather than by carrier status alone, are needed. For counselling, the practical message is that a single severe allele already confers a clinically meaningful cognitive risk, whereas biallelic status implies earlier onset rather than a categorically different disease.

### 2.4. Genetic and Environmental Modifiers of GBA1-Associated Risk

Penetrance of *GBA1* variants is incomplete: most heterozygous carriers never develop a Lewy body disorder, and lifetime risk estimates for Parkinson’s disease or DLB in carriers generally remain below 20%. Modifier biology therefore accounts for a substantial part of the variance in whether, when, and with what phenotype a carrier converts, and it is the most direct route from a non-modifiable risk allele to a modifiable clinical outcome.

The best-supported genetic modifiers act within the same α-synuclein–lysosome axis. A dedicated analysis of *GBA1* carriers with Parkinson’s disease and with Lewy body dementia identified common variation at *SNCA* and at CTSB, which encodes cathepsin B, as modifiers of both risk and age at onset, implicating substrate dosage and an alternative lysosomal protease pathway as the principal buffers of partial GCase deficiency [32]. Variation in TMEM175, encoding a lysosomal potassium channel required for luminal acidification and for normal GCase activity, further modulates synucleinopathy risk and interacts functionally with *GBA1* [33]. *SCARB2*, whose product LIMP-2 traffics GCase from the endoplasmic reticulum to the lysosome, is a plausible modifier by the same logic. Beyond the lysosome, APOE ε4 is the dominant modifier of the cognitive trajectory: it is independently associated with DLB and with the Alzheimer-type co-pathology that accelerates dementia in Lewy body disease, and it is therefore an important determinant of whether a *GBA1* carrier follows a Parkinson-like or a DLB-like course [4,5,6,34,35]. Polygenic risk scores derived from Parkinson’s disease and DLB genome-wide association studies add a further and largely orthogonal layer of modulation [36,37].

Environmental modifiers are far less securely established, and none has been examined specifically in GBA1-mutant DLB. The broader synucleinopathy literature implicates occupational exposure to pesticides, notably paraquat and rotenone, and to chlorinated solvents such as trichloroethylene; both impair mitochondrial complex I and autophagic flux and are therefore plausible amplifiers of a pre-existing lysosomal deficit [38,39]. Conversely, cigarette smoking and caffeine intake are consistently associated with lower Parkinson’s disease risk, although whether these associations are causal, and whether they extend to DLB, remains contested [39]. The one dedicated case–control study of DLB risk factors reported associations with anxiety, depression, a family history of Parkinson’s disease, low caffeine intake, and fewer years of education—a profile that overlaps only partially with the Parkinson’s disease risk factor set [40]. Traumatic brain injury, chronic systemic inflammation, and the gut microbiome have each been proposed as gene–environment intermediates, but the supporting evidence in carriers is anecdotal.

Two methodological points deserve emphasis. First, no adequately powered gene–environment interaction study has been performed in *GBA1* carriers, so the exposures listed above should be regarded as candidate modifiers rather than established ones. Second, several putative environmental modifiers—education, caffeine intake, and depression—are vulnerable to reverse causation because prodromal DLB alters mood, cognition, and habits many years before diagnosis. Prospective carrier cohorts with harmonised exposure assessment, ideally coupled to lysosomal lipid and seed amplification biomarkers, are the necessary next step; the reward would be a modifiable component of risk in a genetically defined and prospectively identifiable population.

### 2.5. Additional Synaptic and Proteostatic Modifiers

Beyond the two principal loci, several genes reinforce the synaptic theme. DNAJC5, encoding cysteine-string protein-α (CSPα), is mechanistically illuminating: CSPα sustains assembly of the SNARE protein SNAP-25, and loss of CSPα produces a neurodegeneration that α-synuclein overexpression can rescue—direct genetic evidence that α-synuclein normally serves a protective presynaptic chaperone function [41]. LRRK2, whose G2019S mutation regulates synaptic-vesicle endocytosis and autophagic flux, contributes to the broader Lewy body disease spectrum, although LRRK2-associated disease frequently shows a lower dementia burden and more variable Lewy pathology [42]. The DLB risk architecture is therefore weighted toward genes governing presynaptic function and lysosomal proteostasis—the two systems summarised in Table 1 and examined next.

## 3. From Lysosomal Dysfunction to Synaptic Plasticity Deficits: The GBA1 Mechanism

### 3.1. Glucocerebrosidase Deficiency and the Sphingolipid–Synuclein Loop

**Figure 3:**
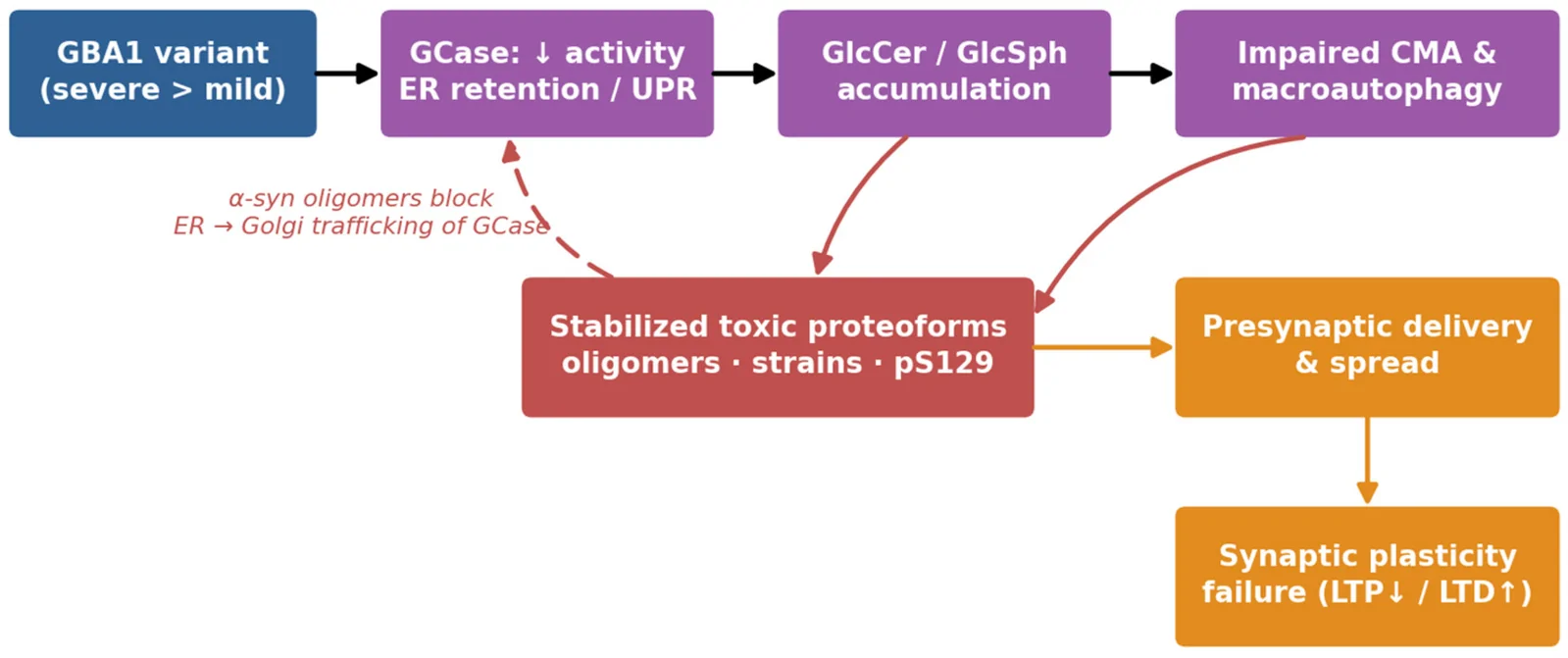
The GBA1–α-synuclein bidirectional loop and the generation of synaptotoxic proteoforms. Loss-of-function *GBA1* variants reduce glucocerebrosidase (GCase) activity and cause endoplasmic reticulum retention, leading to accumulation of glucosylceramide (GlcCer) and glucosylsphingosine (GlcSph). These lipids stabilise toxic α-synuclein proteoforms (oligomers, conformational strains, serine-129-phosphorylated species), which in turn impair the maturation of newly synthesised GCase by blocking its endoplasmic reticulum-to-Golgi trafficking and impair clearance via chaperone-mediated autophagy (CMA), before trafficking to presynaptic terminals to drive plasticity failure.

Glucocerebrosidase hydrolyzes glucosylceramide (GlcCer) to ceramide and glucose within the lysosome. Pathogenic GBA1 variants reduce GCase activity and, for many missense alleles, impair folding and trafficking from the endoplasmic reticulum, triggering ER retention and unfolded-protein-response stress; elevated GlcCer in turn directly stabilises soluble oligomeric intermediates of α-synuclein, lowering the threshold for toxic-species formation [23] (Figure 3). Reduced GCase activity has also been observed in sporadic PD and DLB brain that lacks GBA1 mutations [24,43].

This relationship is bidirectional and self-amplifying: oligomeric α-synuclein impedes ER-to-Golgi trafficking of nascent GCase so that the two defects reinforce each other [23]. The loop is compounded by impairment of α-synuclein clearance through chaperone-mediated autophagy, in which α-synuclein is a LAMP2A-dependent substrate, and macroautophagy [44]. Beyond GlcCer itself, accumulation of glucosylsphingosine and broader lysosomal lipid remodelling further promote aggregation and chaperone-mediated autophagy failure [45,46], as demonstrated in patient-derived GBA1-mutant and knockout neurons [47]. Conversely, pharmacological or genetic restoration of GCase activity reduces pathological α-synuclein and rescues lysosomal function [48], and GCase activity modulates neuronal vulnerability to seeded α-synuclein pathology [49].

### 3.2. Proteoform Diversity as the Molecular Currency of Toxicity

A central conceptual advance is that “α-synuclein” is not a single entity but a spectrum of proteoforms differing in conformation, assembly state, and post-translational modification and that these proteoforms differ profoundly in synaptic toxicity [50]. The aggregation pathway proceeds from natively unfolded monomer through metastable oligomers and protofibrils to mature fibrils; converging evidence identifies the soluble oligomeric and protofibrillar species, rather than the insoluble fibril, as the principal synaptotoxic agents [51,52], a property linked to defined structural features of the toxic oligomer [53]. Superimposed on this assembly state axis is conformational “strain” diversity: structurally distinct, self-templating polymorphs—such as those distinguishing DLB/PD from multiple system atrophy—propagate their own architecture, can be discriminated biochemically, and adopt distinct cryo-electron-microscopy folds [54,55,56]. Post-translational modifications add a further layer, with serine-129 phosphorylation marking the majority of aggregated α-synuclein in Lewy pathology, alongside C-terminal truncation, nitration, and ubiquitination that modulate aggregation and clearance [57,58]. Lysosomal lipid imbalance secondary to GBA1 deficiency biases this landscape toward the more synaptotoxic configurations, linking genotype to the specific species that reach the synapse.

### 3.3. Trafficking to the Synapse and Disruption of Pre- and Postsynaptic Machinery

α-Synuclein is physiologically concentrated at presynaptic terminals, where it senses membrane curvature, binds synaptic vesicles, regulates vesicle-pool size and clustering, and promotes SNARE-complex assembly via VAMP2 [11,59]. Pathogenic proteoforms corrupt this localization: excess or aggregated α-synuclein inhibits exocytosis and neurotransmitter release [60,61], sequesters and redistributes SNARE proteins [62], and abnormally clusters synaptic vesicles through direct membrane and synaptobrevin interactions, restricting their mobility [63,64,65]. The presynaptic terminal thus becomes the primary site of functional lesion well before terminal loss is evident [8,9].

Postsynaptically, oligomeric α-synuclein disrupts glutamate receptor trafficking. It modulates synaptic transmission and impairs LTP through NMDA receptor activation [66], targets specific NMDA receptor subunits to produce striatal synaptic dysfunction and memory deficits [67], and engages cellular prion protein (PrP^c^) coupled to metabotropic glutamate receptor 5 (mGluR5) to drive cognitive impairment via NMDAR2B—a receptor-mediated route independent of pore formation [68]. The combined assault on presynaptic release and postsynaptic receptor handling sets the stage for the failure of bidirectional synaptic plasticity.

## 4. Mechanisms of Plasticity Failure: LTP and LTD

The functional signature of DLB synaptopathy is a metaplastic imbalance: suppression of LTP coupled with facilitation of, or aberrant induction of, LTD (Figure 3). In hippocampal CA1 and cortical preparations, α-synuclein oligomers or excess α-synuclein inhibit LTP at Schaffer-collateral synapses while lowering the threshold for LTD [66,67]. Because this shift is detectable before measurable synapse or neuron loss, it represents an early and potentially reversible stage rather than an epiphenomenon of degeneration [69].

Mechanistically, LTP induction requires NMDA receptor-mediated calcium influx of sufficient amplitude to activate Ca^2+^/calmodulin-dependent protein kinase II and drive AMPA receptor insertion, whereas LTD is favoured by lower-amplitude, sustained calcium signals activating calcineurin and protein phosphatase 1 [66]. By promoting glutamate release from astrocytes and excessive extrasynaptic GluN2B-mediated signalling, α-synuclein oligomers entrench an LTD-favouring, pro-excitotoxic state and degrade the dynamic range over which synapses can be strengthened [68,70]; expression of mutant α-synuclein produces parallel deficits in synaptic targeting and spine stability [71].

Synaptic calcium dysregulation is a recurring node, with oligomers permeabilizing membranes and dysregulating calcium handling at the terminal and spine [51]. This calcium burden converges on the synaptic mitochondrion: dopamine oxidation links mitochondrial and lysosomal dysfunction in a feed-forward cascade [72], and GCase is itself imported into mitochondria where it preserves complex I integrity and energy metabolism so that its deficiency compromises the synaptic ATP supply on which vesicle cycling and receptor trafficking depend [73]. The synapse, with its high energy requirements and dependence on precise calcium signalling, is thereby rendered the most vulnerable neuronal compartment—a vulnerability amplified, rather than created, by the GBA1-driven proteoform shift [74].

## 5. Circuit Dynamics and Neuropsychiatric Phenotypes

The translation of synaptic plasticity failure into the clinical syndrome of DLB requires mapping these deficits onto specific circuits. The cholinergic system is paramount: degeneration of the nucleus basalis of Meynert and cortical cholinergic denervation are more severe in DLB than in Alzheimer’s disease, and the magnitude of cholinergic loss correlates with the severity of cognitive fluctuations and of visual hallucinations [75,76,77]. Acetylcholine is a powerful neuromodulator of cortical and hippocampal plasticity; its depletion compounds the intrinsic glutamatergic deficit and provides the rationale for the symptomatic benefit of cholinesterase inhibitors [78].

**Figure 4:**
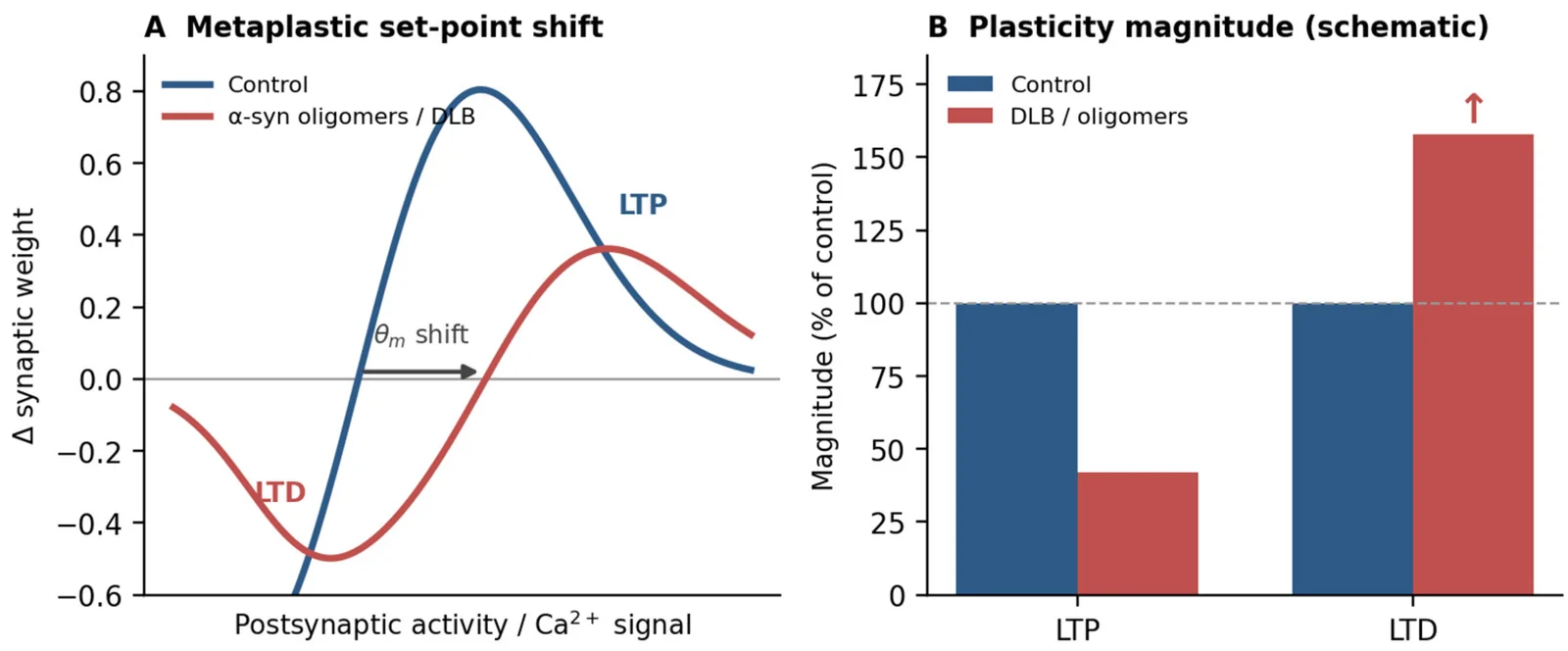
Metaplastic shift underlying synaptic failure in DLB. (**A**) Schematic synaptic-modification (Bienenstock–Cooper–Munro-type) function relating the change in synaptic weight to postsynaptic activity and calcium. α-Synuclein oligomers shift the modification threshold (θ_m_) rightward and compress the potentiation domain. (**B**) Schematic relative magnitude of LTP (reduced) and LTD (enhanced) in the presence of synaptotoxic α-synuclein proteoforms, expressed as a percentage of control. Values are illustrative of the direction and qualitative magnitude reported across model systems. The solid gray line in (**A**) represents the zero-point for synaptic weight change, and the dashed gray line in (**B**) represents the 100% baseline of the control. The gray and red arrows indicate the direction of the pathological shifts.

Cognitive fluctuations are best understood as an emergent property of unstable thalamocortical and cholinergic network dynamics in a plasticity-impaired, cholinergically depleted system [75]. Recurrent visual hallucinations—the most characteristic neuropsychiatric feature—arise from hypofunction of occipital and ventral visual-association cortices, imbalance between dorsal and ventral attentional networks, and cholinergic–dopaminergic dysregulation [79,80]. Within the hippocampal–cortical memory system, the LTP/LTD imbalance degrades encoding and consolidation; hippocampal volume may be relatively preserved early in DLB, underscoring that the deficit is initially functional and synaptic rather than atrophic [9,81]. Framing fluctuations and psychosis as the behavioural readout of genetically initiated, plasticity-level network instability (Figure 4) situates DLB squarely within the neuropsychiatric domain.

## 6. Mechanistic Specificity: What Distinguishes DLB from Other Genetic Synaptopathies

A legitimate objection to the framework developed above is that it may not be specific. Synaptic failure preceding neuronal loss has been documented in Alzheimer’s disease, in frontotemporal lobar degeneration, in Huntington’s disease, and in prion disease, and synapse loss is the strongest neuropathological correlate of cognitive impairment across all of them [7,82]. Familial Alzheimer’s disease caused by PSEN1 and PSEN2 mutations makes the point forcefully because presenilins are not only the catalytic subunit of γ-secretase but also direct regulators of presynaptic neurotransmitter release and of endoplasmic reticulum calcium handling so that PSEN1 mutations generate synaptic dysfunction through both amyloid-dependent and amyloid-independent routes [83,84]. Amyloid-β oligomers, like α-synuclein oligomers, suppress LTP and facilitate LTD, and they do so partly through the same postsynaptic module—cellular prion protein coupled to mGluR5 and Fyn—that mediates α-synuclein oligomer toxicity [68,85,86]. This convergence is real and should be acknowledged rather than argued away.

Specificity resides not in the existence of synaptic failure but in four dissociable features: the initiating genetic lesion, the synaptic compartment affected first, the neurochemical system placed at risk, and the temporal signature of the resulting network instability. First, the proximate molecular species differ. In PSEN1 and PSEN2 disease the initiating event is a shift in γ-secretase processivity that raises the Aβ42:Aβ40 ratio; in DLB it is a lysosomal lipid disturbance that stabilises particular α-synuclein conformers [23,45,87]. These are not interchangeable: glucosylceramide does not generate Aβ oligomers, and no GBA1 variant alters amyloid precursor protein processing. Second, the compartment differs. Alzheimer-type pathology is dendritic and postsynaptic from the outset—tau mislocalises to spines, spine density falls in the entorhinal–hippocampal system, and the earliest deficit is in dendritic integration [88,89]. The DLB lesion is presynaptic: aggregated α-synuclein accumulates at terminals rather than in dendrites, and presynaptic burden, not inclusion count, tracks with cognitive decline [8,9,90].

Third, the neurochemical systems at risk are distinct, and it is here that the genetics becomes clinically legible. α-Synuclein is enriched in, and GCase deficiency is most consequential for, the long, poorly myelinated, high-energy axons of the cholinergic basal forebrain, the brainstem monoaminergic nuclei, and the ventral visual stream. The cholinergic deficit of DLB is accordingly more severe than that of Alzheimer’s disease at comparable dementia severity, and rapid eye movement sleep behaviour disorder, dysautonomia, and well-formed visual hallucinations belong to the Lewy body phenotype rather than to the amyloid one [75,76,77,79,80]. Fourth, the temporal signature differs: an unstable, intermittently failing thalamocortical and cholinergic network produces the cognitive fluctuations that are diagnostic of DLB and are not a feature of PSEN1-associated dementia, whose course is comparatively monotonic [1,75]. The same logic separates DLB from the tauopathies and from Huntington’s disease: MAPT mutations drive dendritic tau mislocalisation and Fyn-dependent excitotoxicity within frontotemporal networks, without cholinergic denervation or rapid eye movement sleep behaviour disorder, whereas mutant huntingtin degrades corticostriatal LTP through extrasynaptic GluN2B signalling and BDNF–TrkB failure in a circuit that produces chorea rather than fluctuating cognition [88,89,91].

These distinctions are relative rather than absolute, and two observations should temper them. Lewy pathology is common in PSEN1 and APP kindreds, in which α-synuclein-positive inclusions occur in a substantial proportion of cases, indicating that amyloidogenic processing can itself promote synuclein aggregation [92]. Reciprocally, Alzheimer-type co-pathology is present in most autopsy-confirmed DLB and independently accelerates dementia onset and shortens survival [34,35]. The genetic architecture reflects this admixture, since APOE ε4 is a risk factor for both diseases and for their combination. DLB is therefore best conceptualised not as a mechanism unique to itself but as a distinct centre of gravity within a shared synaptopathic space—one in which lysosomal lipid-driven α-synuclein proteoforms act first, and most severely, at the presynaptic terminal of cholinergic and visual-network neurons.

This framing is testable rather than merely rhetorical. Seed amplification assays discriminate α-synuclein-driven from amyloid-driven disease in vivo with high sensitivity and specificity, and conformational assays further separate DLB- and Parkinson-type from multiple system atrophy-type strains, which is precisely the specificity that the model predicts [55,93]. On the same account, synaptic positron emission tomography with SV2A ligands should show an earlier and more presynaptically weighted deficit in DLB than in PSEN1-associated Alzheimer’s disease matched for cognitive severity [94]. Table 2 summarises the principal points of convergence and divergence.

## 7. Therapeutic Implications: Restoring Synaptic Health

### 7.1. Targeting the GBA1–α-Synuclein Axis

The mechanistic centrality of the GBA1–α-synuclein loop has made it the most actively pursued disease-modifying target in DLB and GBA-associated PD. Pharmacological chaperones that enhance GCase trafficking and activity are the most advanced strategy: the repurposed mucolytic ambroxol crosses the blood–brain barrier, raises brain GCase activity, and demonstrated central nervous system target engagement in a clinical proof-of-concept study, with reduction in α-synuclein in preclinical models [95,96]. Augmenting CNS GCase—pharmacologically or by adeno-associated viral gene therapy—ameliorates synucleinopathy in animal models [97]. By contrast, substrate reduction therapy with the glucosylceramide synthase inhibitor venglustat showed no benefit in the phase 2 MOVES-PD trial, which tempered expectations and underscored the importance of patient stratification and of treatment timing [98].

### 7.2. Gene Therapy and Enzyme Augmentation: Preclinical Proof and First-in-Human Trials

Because GBA1-associated disease is a partial loss-of-function, restoring enzyme rather than removing substrate is the most mechanistically direct strategy available, and it has the strongest preclinical foundation in this field. In a mouse model of Gaucher-related synucleinopathy, adeno-associated viral delivery of GBA1 to the central nervous system reduced α-synuclein and ubiquitin aggregates and reversed memory deficits [99]. The same approach in α-synuclein-overexpressing mice diminished proteinase-K-resistant α-synuclein and improved behavioural outcomes, and in Gba1 knock-in mice it corrected both glycosphingolipid accumulation and synuclein pathology [97]. In rats, adeno-associated viral expression of glucocerebrosidase in the substantia nigra prevented α-synucleinopathy of midbrain dopamine neurons after an α-synuclein challenge, showing that the protection is neuron-autonomous and not confined to a single species or model [100]. Pharmacological chaperoning with ambroxol raises brain GCase activity and lowers α-synuclein in wild-type and transgenic mice and in non-human primates, providing convergent evidence that the axis is drug-tractable [96].

Clinical translation has begun, although not yet in DLB. LY3884961 (formerly PR001), an adeno-associated virus serotype 9 vector encoding GBA1 and delivered by a single intracisternal injection, has been evaluated in a phase 1/2 study in GBA1-associated Parkinson’s disease (PROPEL) and in a parallel study in neuronopathic type 2 Gaucher disease (PROVIDE); the programme has established the feasibility of cisternal delivery, with hepatic and immunological monitoring driven by the known risks of systemic exposure to adeno-associated viral vectors [101,102]. Small-molecule enzyme augmentation is at a comparable stage: BIA 28-6156 (formerly LTI-291), a non-inhibitory allosteric GCase activator, was safe and increased enzyme activity in a phase 1b study in GBA1-associated Parkinson’s disease and has advanced to phase 2 [103]. Antisense oligonucleotide lowering of SNCA has entered first-in-human study in multiple system atrophy, and the same modality is conceptually applicable to SNCA multiplication carriers and, in principle, to DLB [104].

Three obstacles specific to DLB should be stated plainly. First, no gene therapy or GCase activator trial has yet enrolled a DLB cohort; the closest evidence is a randomised trial of ambroxol in Parkinson’s disease dementia, a phenotype that overlaps DLB by convention rather than by biology [105]. Second, the anatomical target differs: nigrostriatal delivery sufficient for parkinsonism will not reach the widely distributed cortical, limbic, and basal forebrain terminals whose failure produces dementia, so cortical biodistribution rather than nigral transduction should be the primary pharmacological endpoint of a DLB programme. Third, the therapeutic window implied by the synaptopathy model lies before dementia is established, which forces enrolment of prodromal or at-risk GBA1 carriers—identified by seed amplification assays and polysomnography-confirmed rapid eye movement sleep behaviour disorder—and correspondingly forces reliance on biomarker rather than clinical endpoints. Until such trials are undertaken, the honest statement is that gene therapy for GBA1-associated DLB is preclinically well supported, clinically untested, and operationally feasible.

### 7.3. Strategies to Rescue Synaptic Plasticity Directly

Complementary approaches target α-synuclein proteoforms and the synapse itself. Anti-α-synuclein immunotherapies advanced from phase 1 [106] to phase 2 but produced mixed results—cinpanemab failed to meet endpoints, and prasinezumab yielded only partial signals—underscoring the need to engage the precise synaptotoxic proteoform rather than total protein [107,108]. Direct plasticity-restorative strategies include NMDA receptor modulation: memantine conferred modest benefit in randomised trials in DLB and in PD dementia, and selective targeting of extrasynaptic GluN2B-containing receptors remains conceptually attractive [109]. Symptomatically, cholinesterase inhibitors remain the cornerstone of cognitive and neuropsychiatric management, partially compensating for cholinergic modulation of plasticity [110,111], while small molecules that enhance proteostasis seek to deplete toxic species upstream [48]. The overarching logic is to intervene during the synaptic failure window—after genetic risk has been expressed as a plasticity deficit but before degeneration is irreversible.

Table 3 summarises the therapeutic candidates that are most relevant to the GBA1–α-synuclein–synapse axis, together with the most advanced stage of clinical development reached for each, and the principal advantages and limitations of each approach. Two patterns are apparent. Strategies that augment enzyme activity have advanced further and have accumulated more consistent target engagement data than strategies that reduce substrate or that sequester extracellular α-synuclein, and every disease-modifying candidate listed has been tested in Parkinson’s disease rather than in DLB so that the evidence base for the disease under discussion here remains almost entirely extrapolated.

## 8. Conclusions and Future Directions

The genetic architecture of DLB resolves, at the functional level, into an architecture of synaptic failure. SNCA dosage and conformation determine the load and identity of α-synuclein available to the synapse; GBA1 loss-of-function biases the proteoform landscape toward the species most corrosive to synaptic function; and these proteoforms disable SNARE-dependent release and glutamate receptor trafficking to produce a metaplastic collapse of LTP and aberrant facilitation of LTD, which propagates through cholinergic, thalamocortical, and hippocampal–cortical circuits to generate the neuropsychiatric phenotype.

Realising this framework will require multi-omic integration linking genotype to molecular phenotype to circuit function. Polygenic and sequencing studies continue to refine DLB-specific risk and to resolve genetically distinct subgroups [36,37], while single-cell and spatial transcriptomic and proteomic mapping of vulnerable synaptic populations, lipidomic profiling of the GBA1-driven sphingolipid shift, and structural characterisation of disease-specific strains are needed to define which proteoforms drive plasticity failure in which cells [112,113,114,115]. Coupling these molecular maps to functional read-outs—synaptic positron emission tomography, electrophysiological signatures of metaplastic imbalance, and patient-derived neuronal and organoid models—together with synapse-level pathology and receptor-targeted interventions [90,94,116,117], would establish the causal chain end to end and identify biomarkers of the synaptic failure window. Treating the synapse as the integrative endpoint of genetic risk clarifies DLB’s standing as a genetically determined neuropsychiatric synaptopathy and defines the most rational target for early, disease-modifying therapy.

Three priorities follow directly from the analysis presented here. First, DLB cohorts should be genotyped with assays capable of resolving GBA1 complex and recombinant alleles and should be stratified by predicted residual enzyme activity rather than by carrier status so that the dose–phenotype relationship can be tested in DLB itself rather than inferred from Parkinson’s disease. Second, modifier discovery should be extended from risk to conversion, using prospective carrier cohorts in which genetic modifiers, candidate environmental exposures, and lysosomal lipid biomarkers are measured together. Third, the enzyme augmentation and gene therapy programmes that have reached the clinic in GBA1-associated Parkinson’s disease should be extended to prodromal and early DLB, with cortical and limbic biodistribution and synaptic biomarkers as the primary endpoints because the synaptic failure window identified in this review closes before dementia is established.

## Figures and Tables

**Table 1:** Major genetic loci of dementia with Lewy bodies, their encoded synaptic proteins, principal variant classes, and the route by which each converges on synaptic plasticity failure and the neuropsychiatric phenotype.

Locus	Protein and Normal Synaptic Role	Principal Variant Class	Consequence for Synaptic Plasticity	Neuropsychiatric Correlate (Ref.)
SNCA (4q22)	α-Synuclein; presynaptic; promotes SNARE assembly via VAMP2, vesicle clustering and release regulation	Copy-number variants (duplication, triplication); missense (A53T, A30P, E46K, G51D)	Dose- and conformation-dependent oligomer load; impaired SNARE assembly; LTP suppression	Triplication → early dementia and psychosis; marks DLB-end severity [12,13,14,15,16,17]
GBA1 (1q21)	Glucocerebrosidase (GCase); lysosomal glucosylceramide hydrolysis; proteostasis	Heterozygous loss-of-function; severe (p.L483P) vs. mild (p.N409S) alleles	GlcCer-driven stabilisation of toxic proteoforms; lysosomal/autophagic clearance failure; metaplastic shift	Strongest DLB risk factor; severe alleles → faster cognitive decline, florid psychosis [19,20,21,22,23]
DNAJC5/SNARE genes	Cysteine-string protein-α (CSPα) co-chaperone; SNAP-25/VAMP2 release machinery	Loss-of-function and candidate modifier variants	Loss of presynaptic chaperone buffering; instability of the release apparatus	Adult-onset neurodegeneration; candidate plasticity modifiers [11,41]
LRRK2 (12q12)	Leucine-rich repeat kinase 2; synaptic-vesicle endocytosis, autophagic flux	G2019S (gain-of-kinase-function)	Altered vesicle cycling and autophagy; indirect plasticity effects	Lower dementia burden; variable Lewy pathology [42]

LTP, long-term potentiation; GlcCer, glucosylceramide; SNARE, soluble N-ethylmaleimide-sensitive factor attachment protein receptor.

**Table 2:** Convergent and divergent features of genetically defined synaptopathies. All four disorders converge on early synaptic failure, but they differ in the initiating lesion, the compartment affected first, the vulnerable neurochemical system, and the resulting clinical signature.

Disorder (Principal Genes)	Initiating Molecular Lesion	First Compartment Affected	Plasticity Signature	Selectively Vulnerable System	Distinguishing Clinical Marker (Ref.)
DLB (SNCA, GBA1)	Lysosomal GlcCer accumulation stabilising toxic α-synuclein proteoforms	Presynaptic terminal	LTP suppression with facilitated LTD; SNARE and vesicle cycling failure	Cholinergic basal forebrain, brainstem monoaminergic nuclei, ventral visual stream	Cognitive fluctuations, RBD, well-formed visual hallucinations; positive α-synuclein seed amplification [1,8,9,93]
Familial Alzheimer’s disease (PSEN1, PSEN2, APP)	Increased Aβ42:Aβ40 ratio; presenilin-dependent ER Ca^2+^ and release defects	Dendritic spine and postsynaptic density	Aβ-oligomer-driven LTP block and LTD facilitation via PrPc–mGluR5–Fyn; dendritic tau mislocalisation	Entorhinal–hippocampal and default-mode networks	Progressive amnestic decline without fluctuations; amyloid and tau biomarkers [83,85,87]
Frontotemporal lobar degeneration (MAPT)	Mutant tau mislocalisation to dendrites and altered microtubule binding	Dendritic spine	Fyn-dependent excitotoxic signalling; loss of spine stability	Frontal and anterior temporal cortex, von Economo neurons	Behavioural or language syndrome; no cholinergic deficit or RBD [88,89]
Huntington’s disease (HTT)	Polyglutamine-expanded huntingtin; impaired BDNF transport	Corticostriatal synapse (pre- and postsynaptic)	Loss of corticostriatal LTP; extrasynaptic GluN2B-mediated excitotoxicity	Striatal medium spiny neurons	Chorea and progressive dysexecutive syndrome; fully penetrant CAG expansion [91]

Aβ, amyloid-β; BDNF, brain-derived neurotrophic factor; DLB, dementia with Lewy bodies; ER, endoplasmic reticulum; GlcCer, glucosylceramide; LTD, long-term depression; LTP, long-term potentiation; mGluR5, metabotropic glutamate receptor 5; PrPc, cellular prion protein; RBD, rapid eye movement sleep behaviour disorder; SNARE, soluble N-ethylmaleimide-sensitive factor attachment protein receptor.

**Table 3:** Therapeutic candidates relevant to the GBA1–α-synuclein axis and to synaptic rescue in dementia with Lewy bodies, with the most advanced stage of clinical development reached to date. No agent has yet been tested in a dedicated DLB population.

Strategy/Agent	Target and Mechanism	Most Advanced Stage (Population)	Potential Advantages	Limitations and Risks	Ref.
Ambroxol	Pharmacological chaperone; improves GCase folding and lysosomal delivery	Phase 2 completed in GBA1 and non-GBA1 Parkinson’s disease; randomised trial in Parkinson’s disease dementia; phase 3 under way	Oral, blood–brain-barrier penetrant, repurposed and inexpensive; CSF target engagement demonstrated	Enzyme increases are modest and variable; optimal dose and disease stage undefined; no DLB trial	[95,96,105]
Venglustat	Glucosylceramide synthase inhibition (substrate reduction)	Phase 2 completed (MOVES-PD, GBA1-associated Parkinson’s disease)	Oral, with demonstrable peripheral target engagement	Did not meet endpoints and showed unfavourable trends; substrate reduction may be the wrong direction in brain	[98]
BIA 28-6156 (LTI-291)	Non-inhibitory allosteric activator of GCase	Phase 2 in GBA1-associated Parkinson’s disease	Oral; direct enzyme activation independent of variant folding class; good CNS exposure	Efficacy unproven; restricted to GBA1 carriers; no DLB data	[103]
LY3884961 (PR001), AAV9-GBA1	One-time intracisternal gene replacement restoring GCase expression	Phase 1/2 (PROPEL, GBA1-associated Parkinson’s disease; PROVIDE, neuronopathic Gaucher disease)	Potentially durable after a single administration; addresses the primary genetic deficit	Irreversible; immunogenicity and hepatotoxicity risk; uncertain cortical biodistribution; high cost	[101,102]
Prasinezumab, cinpanemab	Monoclonal antibodies against extracellular α-synuclein	Phase 2 completed in early Parkinson’s disease	Target the transmissible extracellular species; well tolerated	Failed primary endpoints; limited CNS penetration; directed at total protein rather than at the synaptotoxic proteoform	[107,108]
ION464 (BIIB101)	Antisense oligonucleotide lowering SNCA expression	Phase 1 in multiple system atrophy	Reduces the pathogenic substrate itself; intrathecal delivery achieves CNS exposure	Loss of physiological α-synuclein may be harmful; repeated invasive dosing; no DLB trial	[104]
Memantine	Uncompetitive NMDA receptor antagonism; preferential block of extrasynaptic receptors	Randomised controlled trials in DLB and Parkinson’s disease dementia; approved in Alzheimer’s disease	Addresses the LTD-favouring, pro-excitotoxic state directly; well tolerated	Small symptomatic effect; not disease-modifying; not selective for extrasynaptic GluN2B	[109]
Rivastigmine, donepezil	Cholinesterase inhibition restoring cholinergic modulation of plasticity	Approved (rivastigmine in Parkinson’s disease dementia; donepezil for DLB in Japan); randomised evidence in DLB	Improve cognition, fluctuations, and hallucinations; established safety in DLB	Symptomatic only; cholinergic adverse effects; no effect on proteoform burden	[110,111]

AAV9, adeno-associated virus serotype 9; CNS, central nervous system; CSF, cerebrospinal fluid; DLB, dementia with Lewy bodies; GCase, glucocerebrosidase; LTD, long-term depression; NMDA, N-methyl-D-aspartate. Development stages are those reached at the time of writing and should be verified against current trial registries.

## Data Availability

No new data were created or analysed in this study. Data sharing is not applicable to this article.
